# Helminth secretome database (HSD): a collection of helminth excretory/secretory proteins predicted from expressed sequence tags (ESTs)

**DOI:** 10.1186/1471-2164-13-S7-S8

**Published:** 2012-12-13

**Authors:** Gagan Garg, Shoba Ranganathan

**Affiliations:** 1 Dept. of Chemistry and Biomolecular Sciences and ARC Centre of Excellence in Bioinformatics, Macquarie University, Sydney NSW 2109, Australia; 2 Dept. of Biochemistry, Yong Loo Lin School of Medicine, National University of Singapore, 8 Medical Drive, Singapore 117597

## Abstract

**Background:**

Helminths are important socio-economic organisms, responsible for causing major parasitic infections in humans, other animals and plants. These infections impose a significant public health and economic burden globally. Exceptionally, some helminth organisms like *Caenorhabditis elegans *are free-living in nature and serve as model organisms for studying parasitic infections. Excretory/secretory proteins play an important role in parasitic helminth infections which make these proteins attractive targets for therapeutic use. In the case of helminths, large volume of expressed sequence tags (ESTs) has been generated to understand parasitism at molecular level and for predicting excretory/secretory proteins for developing novel strategies to tackle parasitic infections. However, mostly predicted ES proteins are not available for further analysis and there is no repository available for such predicted ES proteins. Furthermore, predictions have, in the main, focussed on classical secretory pathways while it is well established that helminth parasites also utilise non-classical secretory pathways.

**Results:**

We developed a free Helminth Secretome Database (HSD), which serves as a repository for ES proteins predicted using classical and non-classical secretory pathways, from EST data for 78 helminth species (64 nematodes, 7 trematodes and 7 cestodes) ranging from parasitic to free-living organisms. Approximately 0.9 million ESTs compiled from the largest EST database, dbEST were cleaned, assembled and analysed by different computational tools in our bioinformatics pipeline and predicted ES proteins were submitted to HSD.

**Conclusion:**

We report the large-scale prediction and analysis of classically and non-classically secreted ES proteins from diverse helminth organisms. All the Unigenes (contigs and singletons) and excretory/secretory protein datasets generated from this analysis are freely available. A BLAST server is available at http://estexplorer.biolinfo.org/hsd, for checking the sequence similarity of new protein sequences against predicted helminth ES proteins.

## Background

According to the World Health Organization, over two billion people are suffering from human helmintasis and many more are at risk worldwide, especially in developing nations [1]. Helmintasis also results in the economic loss of billions of dollars due to damage of crops and livestock every year [2,3]. Besides their role in causing diseases, helminths also provide some protection against autoimmune diseases [4]. Free-living helminths such as *Caenorhabditis elegans *(the most studied helminth till date) serve as models to understand parasitism [5]. In the case of parasitic organisms, excretory/secretory (ES) proteins play an important role during the parasitic infection as these proteins are responsible for the regulation of the host's immune system for parasite survival inside the host. Such important roles played by ES proteins make these proteins attractive targets for the development of therapeutic strategies [6].

With rapid advances in sequencing technologies, sequencing data has been generated on large scale especially in the area of genomics and transcriptomics. Although short reads generated using 454 Roche pyrosequencing is the major sequencing technique used these days for generating transcriptomic data, expressed sequence tags (ESTs) remain the largest resource of helminthic transcriptomic data, with data available for several helminths. dbEST [7], the largest global repository of ESTs, recorded 71,276,166 entries (as on December 1, 2011, release 120111). EST data has been widely used for ES protein prediction in different transcriptomic studies [8,9] but most of the studies do not cover ES proteins comprehensively, especially non-classically secreted ones [10]. Also, it must be noted here that although the helminth proteome is directly affected by the developmental stage-specific expression and indirectly by change/decrease of 3'UTRs with their developmental stages, the data is so sparse in dbEST for some organisms that all available EST data from different stages are pooled together for the data analysis reported here. These mixed datasets have been used before for other nematode transcriptome studies like *S. ratti *studies [11,12]. We have used such a composite *S. ratti *dataset [12] in our previous secretome analysis [13].

In this study, we compiled ESTs for each helminth organism, covering nematodes, trematodes and cestodes and predicted ES proteins encoded by them, followed by functional annotation and therapeutic target analysis. Our earlier large-scale helminth secretome analysis was carried out using EST2Secretome [14] but the study only considered the classically secreted proteins, based on N-terminal secretory signals and covered only parasitic nematodes. Also, the ES protein sequences predicted as a part of this earlier study were not provided to the scientific community. We believe such predicted ES proteins are a valuable resource for understanding host-parasite interactions and for the development of new therapeutic strategies against helminth infections, for further validation using wet lab assays.

Recently we proposed a new bioinformatics workflow [13] for the prediction of classically and non-classically secreted proteins using 454 transcriptomic data of parasitic nematode, *Strongyloides ratti*. In the present study, we applied our workflow with minor modifications to accommodate EST datasets of 78 different helminth species available from dbEST, including those also available from Nematode.net [15], the largest provider of nematode ESTs.

The data were cleaned, assembled into Unigenes (contigs and singletons), which were then translated into proteins. From these putative proteins, ES proteins were predicted using a series of computational tools, which were further verified by sequence similarity to our in-house experimentally-determined parasitic helminth ES protein dataset (detailed in **Materials and methods**). Predicted ES proteins were functionally annotated in terms of similarity to other known proteins, biochemical pathways, protein families and domains. ES proteins were also searched for homologues in human, *C. elegans, Schistosoma mansoni *and *Schistosoma japonicum*. The analysis results are made available to the scientific community *via *the Helminth Secretome Database (HSD) [16] web portal All the Unigenes and ES protein sequence datasets can be browsed in FASTA format and are available for download. A BLAST web service is also provided for researchers to check the similarity of their protein sequences with our predicted ES datasets.

## Materials and methods

### Expressed sequence tags (ESTs) data sets

For this study, EST datasets for different helminth species were downloaded from NCBI dbEST [7] and analysed locally.

### Bioinformatics approach components

**Figure 1:**
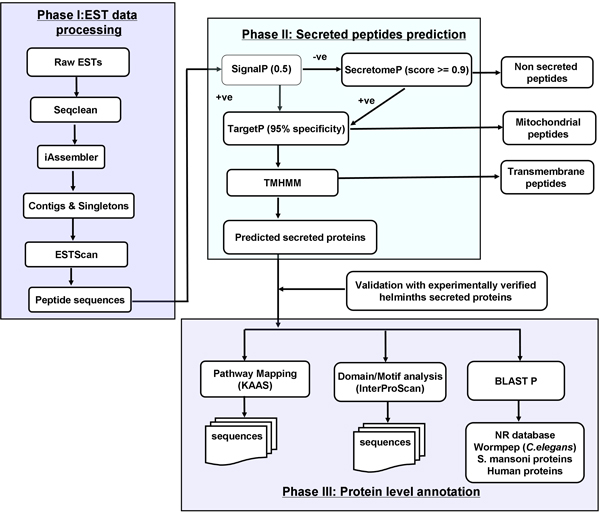
**Secretome analysis workflow based on EST data**. Secretome analysis workflow comprising Phase I (pre-processing and assembly of raw data), II (excretory/secretory (ES) protein prediction) and III (Protein-level functional annotation) based on homologue identification against different databases.

Our bioinformatics approach has three phases as shown in Figure 1, similar to one tested on the *S. ratti *transcriptomic data [13] where we have used MIRA and CAP3 for reliable *de novo *transcriptome assembly, with these tools now combined by a Perl wrapper in iAssembler [17] for the robust assembly of both 454 and Sanger EST datasets. We have implemented our computational approach to the large helminth EST data from dbEST.

Phase I: Preprocessing and assembly of raw EST data

Each organism raw EST data were cleaned to remove short and vector sequences using Seqclean [18] and Univec [19] as a vector database. Seqclean is used to trim and validate ESTs for screening of vector contaminants, low quality and low complexity sequences. Cleaned sequences were assembled using iAssembler (version 1.3.1) [17]. The assembly was carried out using a minimum percent identity for sequence clustering and assembly of 95% contigs and singletons, collectively referred to as Unigenes. ESTScan [20] was used to conceptually translate Unigenes into putative proteins.

Phase II: Prediction and validation of excretory/secretory (ES) proteins

Prediction of ES proteins was carried out using a pipeline of four tools; SignalP [21], SecretomeP [22], TargetP [23] and TMHMM [24] followed by validation with experimentally determined helminth ES proteins as shown in the bioinformatic workflow (Figure 1). This approach of computational prediction of ES proteins has been successfully applied earlier to *Stronglyloides ratti *[13]. SignalP (version 3.0) was used for predicting classically secreted proteins applying options of organism category of eukaryotes and truncation of protein sequence at 70 amino acids. SecretomeP (version 1.0) was used for predicting non-classically secreted proteins using default options. TargetP (version 1.1) was used for the prediction of mitochondrial proteins with a prediction cut-off of 0.78 for mitochondrial protein prediction and 0.73 for other locations. TMHMM (version 2.0) was used for the prediction of transmembrane proteins with default options. Firstly, putative proteins generated from ESTScan were analyzed by SignalP for predicting classically secreted proteins. Proteins were considered secreted, if the D-score and the signal peptide probability computed by SignalP are greater than 0.5. The remaining proteins were then input to SecretomeP for non-classical secretory protein prediction. Proteins were considered as secreted, if the neural network (NN) score from SecretomeP is greater than or equal to 0.9. The combined set of classical and non-classical secretory proteins is then passed to TargetP, to check for mitochondrial proteins. Mitochondrial proteins predicted by TargetP were then removed and the remaining predicted ES proteins analyzed by TMHMM. ES proteins with no transmembrane segments are considered for further analysis.

For the validation of computationally predicted ES proteins, we checked their sequence similarity against our compiled set of 1485 experimentally derived ES proteins of parasitic helminths (*Ancylostoma caninum, Brugia malayi, Clonorchis sinesis, Fasciola hepatica, Schistosoma mansoni, Schistosoma japonicum, Strongyloides ratti *and *Teladorsagia circumcinta) *compiled from literature [25-35] using BLAST [36].

Phase III: ES proteins annotation

Predicted ES proteins from phase II, were annotated for protein domain and family classification using Interproscan [37] including gene ontology (GO) terms option. KAAS [38], provide functional annotation by BLAST comparisons against the manually curated KEGG databases. This tool was used for KEGG pathways BRITE objects mapping [39,40]. ES proteins were independently also searched for homology matching against NCBI's non-redundant protein database and Wormpep (*C. elegans *proteins) [41] using BLAST [36]. ES proteins were also checked for homology matching against human proteins. BLAST was used with permissive (E-value: 1e-05), moderate (1e-15) and/or stringent (1e-30) search strategies. These tools provide fast annotation of large volumes of ES proteins and also reliably used before in other helminth transcriptomic studies [13,14].

### Hardware and Software specifications

The Helminth Secretome database (HSD) is developed using MySQL 5 relational database [42]. The user-friendly interface is developed using PHP [43] for BLAST service and data management. The data is served using the Apache web server [44]. Open source tools used for this study were installed on a ubuntu server operating system based 16-CPU Linux cluster (2.4 GHz, Intel(R) Xeon(R) E5530, 32 RAM). Sequence assembly using iAssembler and protein functional annotation mapping using Interproscan are the most computationally intensive steps.

## Results

Our recently developed bioinformatics workflow applied to 454 transcriptomic dataset of *S. ratti *was modified slightly to be applicable to EST data. The different components of the workflow were linked by Perl, Python and bash shell scripts (Figure 1).

### Preprocessing and assembly of EST datasets

Initially a total of 870,223 ESTs ranging from 59 to 80,905 ESTs for different helminth species were downloaded and stored in different directories on our Linux server. According to the workflow (Figure 1), raw ESTs were cleaned first using Seqclean for removing very short or vector sequences. 846,741 (97.3%) cleaned ESTs were passed to iAssembler for *de novo *assembly. iAssembler is a standalone Perl package to assemble ESTs using iterative cycles of MIRA assemblies followed by CAP3 assembly. The tool gives much higher accuracy in EST assembly than other existing assemblers by employing an iterative assembly strategy and automated error corrections of mis-assemblies [17]. This strategy of using MIRA+CAP3 for *de novo *transcriptome assembly has been successfully implemented earlier for other helminth organisms [13] and therefore, using iAssembler is not only equivalent to these two programs but eliminates an extra step by incorporating the running of both programs in a single step. The assembly results in 303,657 Unigenes, comprising 103,791 contigs and 199,866 singletons. 245,814 proteins were obtained by conceptual translation of Unigenes using ESTScan (Table 1). Statistics of the EST analysis reported here, are provided in Additional file 1: Table S1.

**Table 1:** Summary of EST data analysis

Total number of species	78
Number of Nematode species	64
Number of Trematode species	7
Number of Cestode species	7
Total number of expressed sequence tags (ESTs) analysed	870,223
Total number of Unigenes (contigs + singletons)	303,657
Total number of putative peptides	245,814
Total number of excretory/secretory (ES) proteins predicted	18,992
Total number of ES proteins with annotation	11,390
Total number of ES proteins verified with experimentally derived helminth ES proteins	4,260

### ES protein prediction

Firstly, 18,287 (7.44%) proteins were predicted as classically secreted proteins out of 245,814 total putative proteins using SignalP. The remaining 227,527 (92.56%) putative proteins, predicted to be non-secretory by SignalP, were then scanned by SecretomeP for predicting non-classical secretory proteins. SecretomeP predicted a total of 9,244 (3.76%) non-classically secreted proteins. Combining the results from these two programs yielded a total of 27,531 (11.2%) classical and non-classical proteins which wer then checked by TargetP for identifying mitochondrial proteins. TargetP predicted only 0.17% proteins as mitochondrial, at 95% specificity. The remaining 27,116 proteins after removing 415 mitochondrial proteins were analysed by TMHMM for the prediction of transmembrane proteins. A total of 18,992 (7.72%) proteins were predicted finally as ES proteins after removing 8,126 proteins, which were predicted by TMHMM as transmembrane proteins with at least one transmembrane helix. This number is four fold higher than earlier reported (4710 ES proteins) in the secretome analysis of 39 parasitic nematodes [14].

All ES proteins that were predicted computationally were searched for sequence similarity against our non-redundant dataset of 1,485 experimentally determined ES proteins of various parasitic helminth organisms using BLASTP. We found 4,260 (22.43%) computationally predicted ES proteins homologous to known ES proteins. To the best of our knowledge, the HSD dataset is the most comprehensive collection of ES proteins of helminth organisms. It will serve as a rich source for developing new treatment strategies against parasitic infections and to study the molecular mechanisms of helminth organisms.

### Annotation of ES proteins

ES proteins predicted in Phase II were mapped to known protein families and domains using Interproscan. These proteins were also mapped to biochemical pathways using KAAS. Of the 18,992 ES proteins predicted, we could annotate a total of 7,802 (41.08%) proteins with 2,340 different protein domains and families. ES proteins were annotated with Gene Ontology (GO) terms (2,893 for Biological Process, 4,558 for Molecular Function and 1,588 for Cellular Component) based on Interproscan annotations (species wide annotation available from Additional file 2: Table S2). Table 2 contains the most represented Interpro terms (complete results in Additional file 3: Table S3). Pathway associations were established for 5,893 (31.02%) ES proteins. Maximum number of ES proteins belongs to ***metabolism and human diseases***, making these proteins important in parasitic infections (Table 3). The predicted ES protein dataset comprises important biological molecules, including enzymes, the spliceosome and the ribosome. Table 4 contains the most represented KEGG BRITE objects among the different helminth species (full results available in Additional file 4: Table S4).

**Table 2:** Top 15 most represented domains found in ES proteins using Interproscan

InterPro description	InterPro code	Number of ES proteins (%)
Peptidase C1A, papain	IPR013128	305 (1.60%)
Transthyretin-like	IPR001534	298 (1.57%)
Peptidase C1A, papain C-terminal	IPR000668	276 (1.45%)
CAP domain	IPR014044	267 (1.40%)
Peptidase, cysteine peptidase active site	IPR000169	226 (1.19%)
Allergen V5/Tpx-1-related	IPR001283	204 (1.07%)
Thioredoxin-like fold	IPR012336	190 (1.00%)
C-type lectin fold	IPR016187	170 (0.89%)
Peptidase C1A, cathepsin B	IPR015643	137 (0.72%)
C-type lectin	IPR001304	135 (0.71%)
Metridin-like ShK toxin	IPR003582	127 (0.67%)
Domain of unknown function DUF148	IPR003677	127 (0.67%)
Saposin B	IPR008139	121 (0.64%)
Saposin-like	IPR011001	120 (0.63%)
Glycoside hydrolase, superfamily	IPR017853	120 (0.63%)

**Table 3:** KEGG pathways inferred from predicted ES proteins

Parent KEGG pathway	No. of ESPs	Top KEGG pathway in the category
**Metabolism:**		

Carbohydrate metabolism	296	Citrate cycle (TCA cycle)
Lipid metabolism	221	Fatty acid metabolism
Amino acid metabolism	217	Valine, leucine and isoleucine degradation
Energy metabolism	188	Oxidative phosphorylation
Glycan biosynthesis and metabolism	167	N-Glycan biosynthesis
Nucleotide metabolism	137	Purine metabolism
Xenobiotics Biodegradation and Metabolism	104	Metabolism of xenobiotics by cytochrome P450, Drug metabolism - other enzymes
Metabolism of Cofactors and Vitamins	95	Riboflavin metabolism
Metabolism of other amino acids	70	Glutathione metabolism
Biosynthesis of other Secondary Metabolites	38	Isoquinoline alkaloid biosynthesis
Metabolism of Terpenoids and Polyketides	29	Terpenoid backbone biosynthesis, Limonene and pinene degradation

**Genetic Information processing:**		

Folding, sorting and degradation	446	Protein processing in endoplasmic reticulum
Translation	334	RNA transport
Transcription	176	Spliceosome
Replication and repair	72	Nucleotide excision repair

**Environmental information processing:**		

Signal transduction	243	MAPK signaling pathway
Signalling, molecules and interaction	23	Cell adhesion molecules (CAMs)
Membrane transport	6	ABC transporters

**Cellular processes:**		

Transport and catabolism:	436	Lysosome
Cell Growth and Death	208	Cell cycle
Cell communication	130	Tight junction
Cell Motility	35	Regulation of actin cytoskeleton

**Organismal systems:**		

Immune system	291	Antigen processing and presentation
Nervous System	186	Glutamatergic synapse
Endocrine system	172	Insulin signaling pathway
Digestive System	80	Pancreatic secretion
Circulatory System	52	Cardiac muscle contraction
Excretory System	51	Proximal tubule bicarbonate reclamation
Development	47	Axon guidance
Environmental Adaptation	30	Circadian rhythm - mammal
Sensory System	15	Phototransduction

**Human Diseases:**		

Infectious Diseases	522	HTLV-I infection
Neurodegenerative Diseases	417	Alzheimer's disease
Cancers	241	Pathways in cancer (overview)
Cardiovascular Diseases	55	Hypertrophic cardiomyopathy (HCM), Arrhythmogenic right ventricular cardiomyopathy (ARVC)
Immune Diseases	44	Rheumatoid arthritis
Endocrine and Metabolic Diseases	19	Type II diabetes mellitus

**Table 4:** Top 15 putative functions inferred from predicted ES proteins

BRITE object	No. of species represented (%)
Peptidases	61
Spliceosome	50
Ribosome	49
Transcription Machinery	47
Protein kinases	38
Transfer RNA biogenesis	38
Chaperones and folding catalysts	34
Cytoskeleton proteins	34
Transcription factors	33
Ubiquitin system	26
Translation factors	25
Glycosyltransferases	24
DNA replication proteins	20
Amino acid related enzymes	19
Transporters	18

### Comparative analysis of ES proteins with well-studied organisms

All computationally predicted ES proteins were searched for homology matching against the proteomes of *C. elegans *(Wormpep), *S. mansoni, S. japonicum *and human (Table 5) using BLASTP at an E-value of 1e-05. We also checked for homologues at more stringent E-values (1e-15, 1e -30) (complete results in Additional files 5, 6 and 7). Along with the similarity of our helminth ES protein dataset with other organisms, we checked these proteins for interacting partners based on data obtained from IntAct [45], BioGRID [46] and DIP [47] using BLASTP (interaction results in Additional file 8: Table S8).

**Table 5:** Sequence homology inferred between predicted ES proteins in major helminth organism classes and other well-studied protein datasets at an E-value of 1e-05, using BLASTP

Dataset	Nematode hits	Trematode hits	Cestode hits
*C. elegans *proteins (Wormpep)	8457	345	280
*S. mansoni *proteins	3440	598	419
Human proteins	4539	408	326
NR protein database	10116	652	497
*S. japonicum *proteins	3456	612	416

Our dataset comprises a fairly high number (23, 30%) of parasitic helminth organisms infecting humans so ES proteins were checked for homology matching against the human proteome (Table 5). We found 13,756 (72.4%) ES proteins had no sequence similarity against human proteins and could be preferred targets for parasitic infections. These human dissimilar ES proteins were further searched for sequence similarity against known drug targets available from DrugBank [48]. Of these, 39 ES proteins from human parasitic helminth organisms were found similar to 27 known drug targets and represent potential therapeutic targets. These 27 drug targets are targeted by 75 small drug molecules, out of which 14 are clinically approved drugs. These therapeutic targets are also available from HSD.

### Helminth Secretome database (HSD) data

All the ES proteins and Unigenes generated from this study can be viewed from the HSD data page for each organism. Along with proteins and Unigenes, users have the choice to view protein domain mapping and pathway mapping results. For ES proteins found homologous to known proteins, we provide annotation in the form of sequence identifiers along with percent identity and E-value for BLAST search, e.g. {Acantortus_UN0312; similar to gi|256096002|emb|CAR63732.1| hypothetical protein [Angiostrongylus cantonensis] (Evalue:2e-26, identity:50.00) unverified}. Each annotated ES protein is also tagged as verified or unverified based on the presence or absence of sequence similarity to experimentally determined parasitic helminth ES proteins (Phase II, Figure 1).

### Helminth Secretome database (HSD) BLAST server

**Figure 2:**
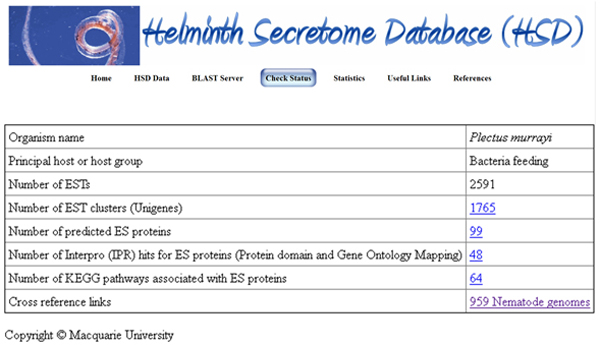
**Screen shot of Helminth Secretome Database (HSD) species page**. Helminth Secretome Database (HSD) species page of *Plectus murrayi*, a bacterial feeding nematode. Users can view Unigenes, ES proteins, protein domain and gene ontology and pathway mapping results from this page.

We have set up a BLAST server to run sequence similarity searches against our predicted ES protein datasets (Figure 2). All ES proteins are divided into three datasets (Nematode ES proteins, Cestode ES proteins and Trematode ES proteins) based on the organism. Users can also query our dataset of experimentally determined helminth ES proteins compiled from literature. The input data uploaded can be either nucleotide or protein sequences in FASTA format. A text box is also provided to paste the sequences directly into the BLAST query submission page. The results from the BLAST search are displayed in HTML format.

## Discussion

Here, we demonstrates the utility of our computational approach, integrating various open source tools, for the prediction and analysis of ES proteins using EST data available from dbEST. All software used in this study are freely available under academic licence. These tools can be installed on different flavours of UNIX based operating system. With the advent of next-generation sequencing (NGS) technologies, there are many transcriptomic studies completed especially for individual helminth species with good coverage but we have focussed on the coverage of a large number of helminth organisms for secretome analysis. The earlier analysis from our group using the EST2Secretome pipeline has now been extended to cover non-classical secretory proteins, with validation against experimentally known excretory/secretory proteins. We plan to carry out further prediction of ES proteins using more comprehensive helminth transcriptomic datasets from NGS platforms and provide the results through HSD.

### Biological implications of this study

Several billion people worldwide are afflicted by infections caused by parasitic helminths. Infections from parasitic helminths, especially from nematodes also results in heavy economic losses worth billions of dollars due to agricultural crop and livestock infection each year. In this study, we have predicted and analysed ES proteins from the largest freely available EST data of several helminth organisms from dbEST.

Many predicted ES proteins map to peptidase domains and families (944,5%) which are reported to be involved in virulence activity (Table 2) and recently, cysteine peptidase expression was studied in a helminth pathogen, *Fasciola hepatica *[49]. Peptidases are well studied in *F. hepatica *for their role in migration and maturation of the parasite within its mammalian host [10]. Another representative Interpro protein domain among the helminth ES proteins is the transthyretin-like domain (1.57%). Transthyretin-like proteins were reported as novel proteins in the *B. malayi *secretome [50]. The most represented functional class among the helminth ES proteins are enzymes, essential for the function of metabolic pathways. Protein kinases, which play a key role in signal transduction, are also present in 38 species of this analysis.

Among the most representative KEGG pathways found in ES proteins are metabolic pathways (8.2%, as shown in Table 3). The top energy metabolism pathway, Oxidative phosphorylation and the top nucleotide metabolism pathway, purine metabolism, found in our pathway analysis were also reported in other helminth transcriptomic studies [13,51]. The second most represented KEGG pathway category among helminth ES proteins are human diseases (6.83%). Association of helminth infections mainly by trematodes with cancers has been recently reviewed [52]. Carcinogenic parasitic trematodes like *Opisthorchis viverrini, Clonorchis sinensis *and *Schistosoma haematobium *were studied in different transcriptomics or genomics studies [53,54].

Representation of ES proteins with immune diseases leads us towards hygiene hypothesis [55]. It is well known that helminth ES proteins modulate the host immune system during the infection for helminth survival inside the host [56]. It is also suggested by regulating the host immune system; helminth species reduce the host susceptibility to allergic and autoimmune diseases [4]. A number of studies are currently underway to test the association of helminth infection with allergic diseases [57]. KEGG pathways contain disease pathways from which we note top neurodegenerative disorder as Alzheimer's disease and top endocrine and metabolic disease as Type II diabetes mellitus (Table 3) in our current ES proteins, which were also found in other helminth transcriptomic studies [13,51]. It is well studied that helminth infection is also associated with diabetes [58,59]. It was hypothesized that helmintic infections may attenuate the development of cardiovascular diseases like atherosclerosis [60]. With the properties of helminth ES proteins for host immune system modulation and involvement of helminth infections in many other disorders, these ES proteins demand further investigation for the development of novel therapeutic strategies. In our attempt to investigate predicted helminth ES proteins as drug targets, we found 27 targets using Drug Bank. Ten *O. viverrini *ES proteins were found similar to β-galactosidase which is used for the development of diagnostic tool for human helminthiasis [61]. *S. stercoralis *ES protein (Sstercoralis_UN2092) was found similar to Cathepsin F. A cathepsin F cysteine protease of *O. viverrini *(human liver fluke) has been characterized [62] and could be a potential therapeutic target as in helminth parasites as this protein is involved in excystation, tissue invasion, catabolism of host proteins for nutrition and immunoevasion [63,64]. We found heme as a potential drug molecule for helminth infection targeting fumarate reductase flavoprotein subunit. This target can be further investigated as helminths lack the heme synthesis pathway [65].

In the present study we have predicted ES proteins from helminth EST data available from dbEST followed by functional annotation of ES proteins in terms of protein domains, pathways and gene ontology and also 39 ES proteins from human parasitic helminth organisms were found similar to known drug targets but it is noteworthy to mention that only few of the targets are validated in helminth organisms. Nearly 40% of predicted ES proteins remain unannotated, which needs to be further investigated using genomic and functional characterization studies.

### Limitations of the current methodology

Integrated computational approaches, similar to those used in this paper, have been applied to other transcriptomic studies [8][13]. These approaches depend on the availability of data for a reference organism from the same taxonomic order. Annotation of the subject organism is based on sequence similarity against proteins present in non-redundant protein database from NCBI and proteins available for well helminth organisms like *C. elegans *(Wormpep), *S. mansoni *and *S. japonicum*. Availability of secretome experimental data is another limiting factor for validation of computationally predicted ES proteins. In the current study, experimentally derived ES proteins from 8 species are used to validate computational predicted ES protein data from 78 species using BLAST. Current validation percentage (22.43%) of computational predicted ES proteins can be further improved by availability of more experimental data. Another limiting factor is that we are predicting functionality based on primary sequence annotation alone, whereas protein function is actually determined by its three dimensional (3D) structure. Therefore, these preliminary predictions of therapeutic targets from this study needs to be further validated using wet-lab assays.

## Conclusion

Our bioinformatics approach made possible the large scale prediction and analysis of ES proteins. As a result of our analysis we develop a unique resource HSD (Helminth Secretome Database) of ES proteins for the parasitology/infectious diseases/pharmacy communities. Our approach can be used on new large-scale transcriptomic data sets from NGS platforms, for rapid prediction and annotation of ES proteins. The approach can be applied to any organism but its main application is for neglected organisms with limited knowledge.

## List of abbreviations used

BRITE: Biomolecular Relations in Information Transmission and Expression; KEGG: Kyoto Encyclopedia of Genes and Genomes; KAAS: KEGG automatic annotation server.

## Competing interests

The authors declare that they have no competing interests.

## Authors' contributions

SR directed the study. GG developed the database and carried out the analysis. SR and GG contributed to writing the manuscript.

## Supplementary Material

Additional File 1 **Summary of large scale helminth EST analysis**. Statistics of excretory/secretory proteins and Unigenes across different helminth species (Table S1) Click here for file

Additional File 2 **Gene Ontology distribution of helminth ES proteins**. Statistics of Gene Ontology distribution across different helminth species (Table S2) Click here for file

Additional File 3 **Helminth ES protein domain mapping**. Represented Interpro domains found in helminth ES proteins. (Table S3) Click here for file

Additional File 4 **KEGG BRITE objects mapping of helminth ES proteins**. Represented KEGG BRITE objects found in ES proteins predicted by KAAS (Table S4) Click here for file

Additional File 5 **Comparison of putative helminth ES proteins with *C. elegans *(Wormpep) and *S. mansoni *proteins**. Statistics of sequence similarity results of helminth ES proteins with *C. elegans *(Wormpep) and *S. mansoni *proteins using BLASTP across different helminth species (Table S5) Click here for file

Additional File 6 **Comparison of putative helminth ES proteins with NR database proteins**. Statistics of sequence similarity results of helminth ES proteins with NR database proteins using BLASTP across different helminth species (Table S6) Click here for file

Additional File 7 **Comparison of putative helminth ES proteins with *S. japonicum*, human proteins**. Statistics of sequence similarity results of helminth ES proteins with *S. japonicum*, human proteins using BLASTP across different helminth species (Table S7) Click here for file

Additional File 8 **Comparison of putative helminth ES proteins with interaction databases proteins**. Statistics of sequence similarity results of helminth ES proteins with interaction databases proteins using BLASTP across different helminth species (Table S8) Click here for file
