# Supplementary material for: Helminth secretome database (HSD): a collection of helminth excretory/secretory proteins predicted from expressed sequence tags (ESTs)
Source: BMC Genomics. 2012 Dec 13;13(Suppl 7):S8. doi: 10.1186/1471-2164-13-S7-S8 (PMC3546426; doi:10.1186/1471-2164-13-S7-S8)
Supplement: Additional File 4 — KEGG BRITE objects mapping of helminth ES proteins. Represented KEGG BRITE objects found in ES proteins predicted by KAAS (Table S4) [file 1471-2164-13-S7-S8-S4.doc]

Additional File 4*:* **Helminth secretome database (HSD): a collection of helminth excretory/secretory proteins predicted from expressed sequence tags (ESTs)**

## Gagan Garg and Shoba Ranganathan

Table S4 - Represented KEGG BRITE objects found in ES proteins predicted by KAAS

| BRITE object | No. of species represented (%) |
| --- | --- |
| Peptidases | 61 |
| Spliceosome | 50 |
| Ribosome | 49 |
| Transcription Machinery | 47 |
| Protein kinases | 38 |
| Transfer RNA biogenesis | 38 |
| Chaperones and folding catalysts | 34 |
| Cytoskeleton proteins | 34 |
| Transcription factors | 33 |
| Ubiquitin system | 26 |
| Translation factors | 25 |
| Glycosyltransferases | 24 |
| [DNA replication proteins](../../../../%5C%5Ckegg-bin%5Cget_htext%3Fq03032.keg+-p+%5Ctools%5Ckaas%5Cfiles%5Clog%5Cresult%5C1326861190) | 20 |
| Amino acid related enzymes | 19 |
| Transporters | 18 |
| Cellular antigens | 18 |
| Proteasome | 17 |
| GTP-binding proteins | 17 |
| Lipid biosynthesis proteins | 16 |
| DNA repair and recombination proteins | 16 |
| Ion Channels | 15 |
| SNAREs | 14 |
| Nuclear receptors | 9 |
| Prenyltransferases | 6 |
| Chromosome | 6 |
| Secretion system proteins | 5 |
| Enzyme-linked receptors | 5 |
| Cytokine receptors | 5 |
| CAM ligands | 4 |
| Cell adhesion molecules (CAMs) | 3 |
| G Protein-Coupled Receptors | 2 |
