# Supplementary material for: Helminth secretome database (HSD): a collection of helminth excretory/secretory proteins predicted from expressed sequence tags (ESTs)
Source: BMC Genomics. 2012 Dec 13;13(Suppl 7):S8. doi: 10.1186/1471-2164-13-S7-S8 (PMC3546426; doi:10.1186/1471-2164-13-S7-S8)
Supplement: Additional File 7 — Comparison of putative helminth ES proteins with S. japonicum, human proteins. Statistics of sequence similarity results of helminth ES proteins with S. japonicum, human proteins using BLASTP across different helminth species (Table S7) [file 1471-2164-13-S7-S8-S7.doc]

Additional File 7 *:* **Helminth secretome database (HSD): a collection of helminth excretory/secretory proteins predicted from expressed sequence tags (ESTs)**

## Gagan Garg and Shoba Ranganathan

Table S7- Statistics of sequence similarity results of helminth ES proteins with interaction databases proteins using BLASTP (at different E values) across different helminth species

| Organism  name | Principal host or host group | Number of interaction databases hits (1e-05) | Number of interaction databases hits (1e-15) | Number of interaction databases hits (1e-30) |
| --- | --- | --- | --- | --- |
| **Trematodes** | | | | |
| *Echinostoma paraensei* | Human | 4 | 2 | 0 |
| *Clonorchis sinensis* | Human | 295 | 188 | 99 |
| *Fasciola gigantica* | Mammals | 75 | 50 | 30 |
| *Fasciola hepatica* | [herbivorous](http://en.wikipedia.org/wiki/Herbivory) [mammals](http://en.wikipedia.org/wiki/Mammal), including humans | 11 | 8 | 3 |
| **Opisthorchis felineus** | Mammals | 65 | 62 | 50 |
| *Opisthorchis viverrini* | Human | 55 | 40 | 25 |
| *Paragonimus westermani* | Human | 11 | 9 | 8 |
| **Cestodes** | | | | |
| *Echinococcus granulosus* | Mammals** | 93 | 50 | 28 |
| *Echinococcus multilocularis* | Mammals** | 23 | 14 | 9 |
| *Mesocestoides corti* | Birds, Mammals including human | 6 | 2 | 1 |
| *Moniezia expansa* | Ruminants (sheep, goat and cattle) | 40 | 26 | 9 |
| *Spirometra erinaceieuropaei* | Human, domestic animals | 66 | 44 | 30 |
| *Taenia saginata* | Cattle, Human | 5 | 4 | 2 |
| *Taenia solium* | Pig, Human | 181 | 108 | 55 |
| **Nematodes** | | | | |
| *Aphelenchus avenae* | Fungus | 114 | 74 | 33 |
| *Ancylostoma caninum* | Dog | 713 | 507 | 300 |
| *Angiostrongylus cantonensis* | Rat | 48 | 37 | 25 |
| *Ancylostoma ceylanicum* | Human, Dog and cat | 215 | 153 | 107 |
| *Ascaris lumbricoides* | Human | 23 | 13 | 0 |
| *Ascaris suum* | Pig | 253 | 219 | 123 |
| *Anisakis simplex* | Human | 8 | 7 | 3 |
| *Anguina tritici* | Plant | 0 | 0 | 0 |
| *Brugia malayi* | Human | 218 | 140 | 76 |
| *Bursaphelenchus mucronatus* | Trees | 131 | 95 | 39 |
| *Bursaphelenchus xylophilus* | Pine wood | 320 | 225 | 122 |
| *Caenorhabditis brenneri* | Bacteria feeding | 691 | 634 | 526 |
| *Caenorhabditis briggsae* | Free living | 60 | 54 | 34 |
| *Caenorhabditis japonica* | Bacteria feeding | 697 | 647 | 545 |
| *Caenorhabditis remanei* | Bacteria feeding | 531 | 493 | 433 |
| *Ditylenchus africanus* | Peanut | 19 | 15 | 6 |
| *Dirofilaria immitis* | Dog | 35 | 21 | 10 |
| *Dictyocaulus viviparus* | Cattle, deer | 85 | 54 | 38 |
| *Globodera pallida* | Tomato,eggplant | 98 | 69 | 37 |
| *Globodera rostochiensis* | Potatoes, Tomato and other plants | 113 | 79 | 48 |
| *Haterorhabditis bacteriophora* | Insects | 400 | 339 | 255 |
| *Haemonchus contortus* | Sheep, goat (small ruminants) | 355 | 271 | 187 |
| *Heterodera glycines* | Soybean | 256 | 170 | 101 |
| *Heterodera schachtii* | Sugar beet, cabbage, cauliflower, brussel sprouts, mustard, radish, spinach, chard | 43 | 29 | 15 |
| *Loa loa* | Human | 30 | 18 | 13 |
| *Litomosoides sigmodontis* | Rodents | 52 | 35 | 27 |
| *Meloidogyne arenaria* | Peanut, vegetables, grasses, fruit, ornamentals and tobacco | 61 | 47 | 25 |
| *Meloidogyne chitwoodi* | Plants like potato, barley, wheat and alfalfa | 79 | 61 | 34 |
| *Meloidogyne incognita* | Cotton, tobacco, peanut and fibre crops | 161 | 112 | 60 |
| *Meloidogyne hapla* | Approximately 500 plant hosts | 173 | 127 | 78 |
| *Meloidogyne javanica* | More than 770 plant hosts | 73 | 51 | 29 |
| *Meloidogyne paranaensis* | Coffee | 37 | 27 | 15 |
| *Necator americanus* | Human | 58 | 41 | 13 |
| *Nippostrongylus brasiliensis* | Rat | 136 | 100 | 65 |
| *Oesophagostomum dentatum* | Pig | 19 | 5 | 3 |
| *Onchocerca flexuosa* | Deer | 13 | 10 | 8 |
| *Onchocerca ochengi* | Cattle | 1 | 1 | 1 |
| *Ostertagia ostertagi* | Cattle | 120 | 77 | 46 |
| *Onchocerca volvulus* | Human | 158 | 120 | 74 |
| *Plectus murrayi* | Bacteria | 74 | 61 | 49 |
| *Pristionchus pacificus* | Free living, bacterial feeding | 297 | 216 | 139 |
| *Pratylenchus penetrans* | Apple, cherry, peach | 15 | 9 | 3 |
| *Panagrolaimus superbus* | Free living | 114 | 79 | 44 |
| *Parelaphostrongylus tenuis* | Deer | 3 | 2 | 2 |
| *Parastrongyloides trichosuri* | Possum | 93 | 69 | 43 |
| *Pratylenchus vulnus* | Fruit trees | 26 | 22 | 13 |
| *Rotylenchulus reniformis* | Plants* | 10 | 5 | 3 |
| *Radopholus similis* | Banana, citrus | 62 | 37 | 17 |
| *Steinernema carpocapsae* | Insects | 30 | 25 | 17 |
| *Steinernema feltiae* | Insects | 3 | 3 | 2 |
| *Strongyloides ratti* | Rat | 97 | 75 | 48 |
| *Strongyloides stercoralis* | Human | 111 | 85 | 57 |
| *Strongyloides venezuelensis* | Rat | 2 | 2 | 1 |
| *Toxocara canis* | Dog | 69 | 37 | 15 |
| *Teladorsagia circumcinta* | Sheep, goat (small ruminants) | 207 | 146 | 92 |
| *Toxascaris leonina* | Dog, cat and fox | 21 | 11 | 7 |
| *Trichuris muris* | Rodent | 84 | 47 | 26 |
| *Trichinella pseudospiralis* | Birds | 87 | 55 | 27 |
| *Trichinella spiralis* | Mammals | 253 | 165 | 87 |
| *Trichostrongylus vitrinus* | Sheep | 10 | 3 | 0 |
| *Trichuris vulpis* | Canids | 48 | 23 | 9 |
| *Wuchereria bancrofti* | Human | 55 | 37 | 27 |
| *Xiphinema index* | Grape | 153 | 102 | 58 |
| *Zeldia punctata* | Free living | 4 | 2 | 2 |

* includes fruit trees, lentil, cotton, cowpea, pigeonpea, tea, tobacco, soybean, pineapple, bananas, okra, coconut, cabbage, sweet potato, alfalfa, corn, asparagus, palm, cucumber, tomato, pumpkin, squash, cassava, radish, eggplant, guava, melon and ginger

** includes [wolves, foxes, jackals, coyotes, domestic dogs](http://en.wikipedia.org/wiki/Canidae) and humans
