# Supplementary material for: Helminth secretome database (HSD): a collection of helminth excretory/secretory proteins predicted from expressed sequence tags (ESTs)
Source: BMC Genomics. 2012 Dec 13;13(Suppl 7):S8. doi: 10.1186/1471-2164-13-S7-S8 (PMC3546426; doi:10.1186/1471-2164-13-S7-S8)
Supplement: Additional File 8 — Comparison of putative helminth ES proteins with interaction databases proteins. Statistics of sequence similarity results of helminth ES proteins with interaction databases proteins using BLASTP across different helminth species (Table S8) [file 1471-2164-13-S7-S8-S8.doc]

Additional File 8*:* **Helminth secretome database (HSD): a collection of helminth excretory/secretory proteins predicted from expressed sequence tags (ESTs)**

## Gagan Garg and Shoba Ranganathan

Table S8- Predicted therapeutic targets from predicted ES proteins of parasitic helminth organisms infecting humans with no homologue in the host, human and homologous to known drug targets in DrugBank.

| No. | Cluster ID  (sequence length) | Description from NR database | Interpro hits | *C. elegans* gene ontology | Interaction partner | DrugBank targets hits | Small drug molecules | Verified with known ES protein |
| --- | --- | --- | --- | --- | --- | --- | --- | --- |
| Trematodes | | | | | | | | |
| 1 | Fgigantica_UN1535 | malate dehydrogenase [Schistosoma mansoni] (Evalue:1e-49, identity:70.08) | IPR003767 | Molecular Function: oxidoreductase activity (GO:0016491)  Biological Process: oxidation-reduction process (GO:0055114) | Hypothetical_protein_CBG12370_[Caenorhabditis_briggsae] { Evalue:2e-32, identity:53.78} | Dehydrogenase | **Nicotinamide-Adenine-Dinucleotide** | yes |
| 2 | Fgigantica_UN2703 | dopamine-beta-monooxygenase [Schistosoma mansoni] (Evalue:5e-09, identity:52.83) | IPR000945  IPR008977 | Molecular Function: dopamine beta-monooxygenase activity (GO:0004500)  Biological Process: oxidation-reduction process (GO:0055114) | copper_type_II,_ascorbate-dependent_monooxygenase,_putative_[Ixodes_scapularis] { Evalue:3e-07, identity:34.38} | Dopamine beta-hydroxylase | **Vitamin C**; **Disulfiram**; **Dopamine (approved)** | No |
| 3 | Ofelineus_UN080  (Hypothetical protein) | No annotation | IPR007856  IPR008139  IPR011001 | Biological Process: lipid metabolic process (GO:0006629) | No interacting partner | Proactivator polypeptide | **Di-Stearoyl-3-Sn-Phosphatidylethanolamine** | No |
| 4 | Oviverrini_UN2048, UN0180, UN0195, UN0201, UN0213, UN0464, UN0465, UN2007, UN2071, UN2086 | beta-galactosidase a-peptide [Cloning vector pTriplEx2] | IPR000683  IPR001155  IPR012615  IPR008979 |  | beta-galactosidase_alpha_polypeptide_[Cloning_vector_pTriplEx] | Beta-galactosidase | **Dimethyl sulfoxide (approved)**  **1-(Isopropylthio)-Beta-Galactopyranside**  **D-Galctopyranosyl-1-On**  **1-O-[O-Nitrophenyl]-Beta-D-Galactopyranose**  **2-Fluoro-2-Deoxy-Beta-D-Galactopyranose**  **(5r,6s,7s,8s)-5-Hydroxymethyl-6,7,8-Trihydroxy-Tetrazolo[1,5-a]Piperidine**  **1-O-[P-Nitrophenyl]-Beta-D-Galactopyranose**  **Allolactose**  **2-Deoxy-Beta-D-Galactose**  **S,S-(2-Hydroxyethyl)Thiocysteine** | No |
| Cestodes | | | | | | | | |
| 5 | Egranulosus_UN0139, UN0390, UN1219, Emultilocaris_UN0174, Tsolium_UN5206 | Hypothetical protein [Taenia solium] | IPR001602 |  | CG31688,_isoform_C_[Drosophila_melanogaster] | BH3498 protein | **Acetic acid (approved)** | No |
| 6 | Serinaceieuropaei_UN1388 | SJCHGC02198 protein [Schistosoma japonicum] | IPR001584  IPR012337 | Molecular Function: DNA binding (GO:0003677), Biological Process: DNA integration (GO:0015074) | PREDICTED:_similar_to_polyprotein_[Danio_rerio] | Pol polyprotein | **N-(4-{[amino(imino)methyl]amino}butyl)-2,4'-bi-1,3-thiazole-4-carboxamide**  **N-1H-imidazol-2-yl-N'-[4-(1H-imidazol-2-ylamino)phenyl]benzene-1,4-diamine** | No |
| 7 | Serinaceieuropaei_UN1828 | procollagen-lysine, 2-oxoglutarate 5-dioxygenase 3 [Schistosoma japonicum] |  |  | PREDICTED:_similar_to_lysyl_hydroxylase,_partial_[Ornithorhynchus_anatinus] | Procollagen-lysine,2-oxoglutarate 5-dioxygenase | **Vitamin C**  **Succinic acid**  **(both approved)** | No |
| 8 | Tsolium_UN5517 | phosphate acetyl/butaryl transferase [Escherichia coli M863] | IPR002505 | Biological Process: metabolic process (GO:0008152), Molecular Function: acyltransferase activity (GO:0008415) | predicted_phosphotransacetylase_subunit_[Escherichia_coli_str._K-12_substr._MG1655] | Glucose-1-phosphatase | **Alpha-D-Glucose-1-Phosphate** | No |
| Nematodes | | | | | | | | |
| 9 | Aceylanicum_UN0876 | putative Lipid Binding Protein [Angiostrongylus cantonensis] | IPR000463  IPR011038 | Molecular Function: transporter activity (GO:0005215), Biological Process: transport (GO:0006810), Molecular Function: lipid binding (GO:0008289) | Lipid_Binding_Protein_family_member mber_(lbp-1)_[Caenorhabditis_elegans] | Myelin P2 protein | **S- Hydroxycysteine**  **Palmitic Acid**  **Lauryl Dimethylamine-N-Oxide**  **Oleic Acid** | yes |
| 10 | Aceylanicum_UN1029 | Hypothetical protein CBG23341 [Caenorhabditis briggsae] | IPR002172 | Molecular Function: protein binding (GO:0005515) | Hypothetical_protein_CBG23341_[Caenorhabditis_briggsae] | Low-density lipoprotein receptor | **Porfimer (approved)**  **Alpha-D-Mannose** | yes |
| 11 | Aceylanicum_UN3131 | CBR-GEI-7 protein [Caenorhabditis briggsae AF16] | IPR000918  IPR001465  IPR011076  IPR01581 | Molecular Function: malate synthase activity (GO:0004474), Biological Process: glyoxylate cycle (GO:0006097) | GEX_Interacting_protein_family_member_(gei-7)_[Caenorhabditis_elegans] | Isocitrate lyase | **Glyoxalate, Glyoxylate** | No |
| 12 | Bmalayi_UN01195 | predicted pyruvate formate lyase [Escherichia coli str. K-12 substr. MG1655] | IPR004184 | Cellular Component: cytoplasm (GO:0005737), Biological Process: glucose metabolic process (GO:0006006), Molecular Function: formate C-acetyltransferase activity (GO:0008861) | Predicted_pyruvate_formate_lyase_[Escherichia_coli_str._K-12_substr._MG1655] | Glycerol dehydratase | **1,2-Propanediol** | No |
| 13 | Bmalayi_UN04283 | Sensor histidine kinase DpiB [Escherichia albertii TW07627] |  |  | sensory_histidine_kinase_in_two-component_regulatory_system_with_citB_[Escherichia_coli_str._K-12_substr._MG1655] | Sensor kinase citA | **Heptamolybdate**  **Mo(Vi)(=O)(Oh)2** | No |
| 14 | Bmalayi_UN09455 | cyclophilin-type peptidyl-prolyl cis-trans isomerase-13, Bmcyp-13 [Brugia malayi] | IPR015891 |  | C._briggsae_CBR-CYN-13_protein_[Caenorhabditis_briggsae] | Peptidyl-prolyl cis-trans isomerase, mitochondrial | **L-Proline (approved)**  **Dimethyl sulfoxide (approved)**  **Methoxy-2-[2-(2-Methoxy-Ethoxy]-Ethane**  **7-AMINO-4-METHYL-CHROMEN-2-ONE** | Yes |
| 15 | Lloa_UN0780 | trypsin 5G1 [Culex quinquefasciatus] | IPR001254  IPR01811  IPR009003 | Molecular Function: serine-type endopeptidase activity (GO:0004252), Biological Process: proteolysis (GO:0006508) | CG14642,_isoform_B_[Drosophila_melanogaster] | Myeloblastin | **Fucose** | No |
| 16 | Namericanus_UN0697 | Ubiquinol-Cytochrome c oxidoReductase complex family member (ucr-2.2) [Caenorhabditis elegans] | IPR007863  IPR011249  IPR001134  IPR00182  IPR00899 | Molecular Function: metalloendopeptidase activity (GO:0004222), Biological Process: proteolysis (GO:0006508), Molecular Function: zinc ion binding (GO:0008270) |  | Cytochrome b-c1 complex subunit 2, mitochondrial | **2-Hexyloxy-6-Hydroxymethyl-Tetrahydro-Pyran-3,4,5-Triol**  **5-n-undecyl-6-hydroxy-4,7-dioxobenzothiazole**  **METHYL (2Z)-2-(2-{[6-(2-CYANOPHENOXY)PYRIMIDIN-4-YL]OXY}PHENYL)-3-METHOXYACRYLATE**  **(5S)-3-ANILINO-5-(2,4-DIFLUOROPHENYL)-5-METHYL-1,3-OXAZOLIDINE-2,4-DIONE**  **FAMOXADONE**  **METHYL (2Z)-3-METHOXY-2-{2-[(E)-2-PHENYLVINYL]PHENYL}ACRYLATE** | No |
| 17 | Namericanus_UN1021 | Abnormal cell migration protein 6, isoform b, partially confirmed  by transcript evidence [Caenorhabditis elegans] | IPR002223  IPR02090 | Molecular Function: serine-type endopeptidase inhibitor activity (GO:0004867) |  | Amyloid beta A4 protein | **Methionine Sulfoxide** | Yes |
| 18 | Ovolvulus_UN0760 | hypothetical protein LOAG_04392 [Loa loa] | IPR008027 | Cellular Component: mitochondrial envelope (GO:0005740), Biological Process: mitochondrial electron transport, ubiquinol to cytochrome c (GO:0006122), Molecular Function: ubiquinol-cytochrome-c reductase activity (GO:0008121) | Cytochrome b-c1 complex subunit 9 |  | **2-Hexyloxy-6-Hydroxymethyl-Tetrahydro-Pyran-3,4,5-Triol**  **5-n-undecyl-6-hydroxy-4,7-dioxobenzothiazole**  **METHYL (2Z)-2-(2-{[6-(2-CYANOPHENOXY)PYRIMIDIN-4-YL]OXY}PHENYL)-3-METHOXYACRYLATE**  **5-HEPTYL-6-HYDROXY-1,3-BENZOTHIAZOLE-4,7-DIONE**  **ANILINO-5-(2,4-DIFLUOROPHENYL)-5-METHYL-1,3-OXAZOLIDINE-2,4-DIONE**  **(5S)-3-ANILINO-5-(2,4-DIFLUOROPHENYL)-5-METHYL-1,3-OXAZOLIDINE-2,4-DIONE**  **FAMOXADONE**  **METHYL (2Z)-3-METHOXY-2-{2-[(E)-2-PHENYLVINYL]PHENYL}ACRYLATE**  **2-NONYL-4-HYDROXYQUINOLINE N-OXIDE**  **UBIQUINONE-2** | No |
| 19 | Ovolvulus_UN2296 | Orn/Lys/Arg decarboxylase, major domain protein [Escherichia coli MS 187-1] | IPR000310  IPR008286  IPR015424 | Molecular Function: catalytic activity (GO:0003824) | Biodegradative_arginine_decarboxylase_[Escherichia_coli_str._K-12_substr._MG1655] | Ornithine decarboxylase, inducible | **Guanosine-5'-Triphosphate** | No |
| 20 | Ovolvulus_UN3521 | adenosylmethionine-8-amino-7-oxononanoate transaminase BioA [Escherichia coli UMNK88] | IPR005814  IPR005815  IPR015424 | Molecular Function: adenosylmethionine-8-amino-7-oxononanoate transaminase activity (GO:0004015), Biological Process: biotin biosynthetic process (GO:0009102) | 7,8-diaminopelargonic_acid_synthase,_PLP-dependent_[Escherichia_coli_str._K-12_substr._MG1655] | Adenosylmethionine-8-amino-7-oxononanoate aminotransferase | **7-Keto-8-Aminopelargonic Acid**  **Amino-4-(4-Amino-Cyclohexa-2,5-Dienyl)-Butyric Acid**  **N'-Pyridoxyl-Lysine-5'-Monophosphate** | No |
| 21 | Ovolvulus_UN3760 | predicted DNA-binding transcriptional regulator [Escherichia coli str. K-12 substr. MG1655] | IPR000847 | Molecular Function: sequence-specific DNA binding transcription factor activity (GO:0003700), Biological Process: regulation of transcription, DNA-dependent (GO:0006355) | Predicted_DNA-binding_transcriptional_regulator_[Escherichia_coli_str._K-12_substr._MG1655] | Hydrogen peroxide-inducible genes activator | **Benzoic Acid** | No |
| 22 | Sstercoralis_UN0659 | hypothetical protein F58H1.7 [Caenorhabditis elegans] | IPR002172 | Molecular Function: protein binding (GO:0005515) | hypothetical_protein_F58H1.7_[Caenorhabditis_elegans] | Low-density lipoprotein receptor-related protein 1 | **Antihemophilic Factor Tenecteplase**  **Coagulation Factor IX**  All biotech approved drugs | Yes |
| 23 | Sstercoralis_UN2092 | CRE-TAG-196 protein [Caenorhabditis remanei] | IPR013128  IPR01320 | Molecular Function: cysteine-type peptidase activity (GO:0008234) | Temporarily_Assigned_Gene_name_family_member_(tag-196)_[Caenorhabditis_elegans] | Cathepsin F | **[1-(1-Methyl-4,5-Dioxo-Pent-2-Enylcarbamoyl)-2-Phenyl-Ethyl]-Carbamic Acid Benzyl Ester**  **[1-(1-Benzyl-3-Hydroxy-2-Oxo-Propylcarbamoyl)-2-Phenyl-Ethyl]-Carbamic Acid Benzyl Ester**  **4-Morpholin-4-Yl-Piperidine-1-Carboxylic Acid [1-(3- Benzenesulfonyl-1-Propyl-Allylcarbamoyl)-2-Phenylethyl]-Amide**  **Benzoyl-Arginine-Alanine-Methyl Ketone**  **WRR-99**  **WRR-112**  **HOMOPHENYLALANINYLMETHANE**  **BENZOYL-TYROSINE-ALANINE-METHYL KETONE** | Yes |
| 24 | Sstercoralis_UN2761 | Serine/threonine-protein kinase H1 [Ascaris suum] | IPR011009  IPR020636 | Molecular Function: transferase activity, transferring phosphorus-containing groups (GO:0016772) | Hypothetical_protein_CBG22088_[Caenorhabditis_briggsae] | Calcium/calmodulin-dependent protein kinase type 1D | **N-(5-METHYL-1H-PYRAZOL-3-YL)-2-PHENYLQUINAZOLIN-4-AMINE** | No |
| 25 | Tspiralis_UN1020 | cleavage and polyadenylation specificity factor subunit 2 [Trichinella spiralis] | IPR003953 | Molecular Function: electron carrier activity (GO:0009055), Molecular Function: oxidoreductase activity (GO:0016491) | Hypothetical_protein_CBG05192_[Caenorhabditis_briggsae] | Fumarate reductase flavoprotein subunit | **Fumarate**  **Heme**  **Flavin-Adenine Dinucleotide**  **Malate Like Intermediate**  **Citraconic acid** | Yes |
| 26 | Tspiralis_UN4092 | ribonucleotide reductase R2 subunit [Trichinella spiralis] | IPR000358 | Molecular Function: ribonucleoside-diphosphate reductase activity (GO:0004748), Biological Process: deoxyribonucleoside diphosphate metabolic process (GO:0009186), Biological Process: oxidation-reduction process (GO:0055114) |  | Ribonucleoside-diphosphate reductase M2 subunit | **Cladribine**  **Gallium nitrate**  **(both approved)** | No |
